# Comparison of Short-Term Effects of Extracorporeal Shock Wave Therapy, Low-Level Laser Therapy and Pulsed Electromagnetic Field Therapy in Knee Osteoarthritis: A Randomized Controlled Study

**DOI:** 10.3390/jcm14020594

**Published:** 2025-01-17

**Authors:** Tugce Pasin, Bilinc Dogruoz Karatekin

**Affiliations:** Department of Physical Medicine and Rehabilitation, Goztepe Prof Dr Suleyman Yalcin City Hospital, 34722 Istanbul, Turkey; tugcepasin@hotmail.com

**Keywords:** extracorporeal shock wave therapy, low-level laser therapy, pulsed electromagnetic field therapy, knee osteoarthritis

## Abstract

**Background:** Knee osteoarthritis (OA) is the most prevalent form of osteoarthritis and a leading cause of chronic pain in adults. This study aimed to compare the short-term effects of extracorporeal shock wave therapy (ESWT), low-level laser therapy (LLLT), and pulsed electromagnetic field therapy (PEMF) on pain, function, and quality of life in patients with knee OA. **Methods:** A hundred and twenty patients with Kellgren–Lawrence grade 2–3 knee OA were randomized into four groups: ESWT (once a week for three sessions), LLLT (twice a week for eight sessions), PEMF (twice a week for eight sessions), and a control group with 30 patients in each group. All participants were instructed in a daily exercise program, including knee joint range of motion, stretching, and strengthening exercises (3 × 10 repetitions). Outcome measures, including the visual analog scale (VAS), the Western Ontario and McMaster Universities Osteoarthritis Index (WOMAC), Short Form-36 (SF-36), and the Timed Up and Go (TUG) test, were assessed at baseline after treatment and at the third month. **Results:** There were no significant differences between groups at baseline regarding VAS, WOMAC, SF-36, and TUG scores (*p* > 0.05). Significant improvements were observed in all parameters post-treatment for all groups (*p* < 0.001). However, the improvements in the PEMF group were significantly lower than in the ESWT and LLLT groups, particularly for VAS, WOMAC pain, and SF-36 physical function scores (*p* < 0.05). No significant differences were found between ESWT and LLLT (*p* > 0.05). **Conclusions:** In the short-term, ESWT, LLLT, and PEMF effectively reduce pain, improve physical function, and enhance quality of life in patients with knee OA, though PEMF showed less pronounced improvements.

## 1. Introduction

Osteoarthritis (OA) is a chronic, degenerative joint disease marked by the breakdown of cartilage, osteophyte formation, and subchondral bone changes. Synovial inflammation (synovitis) plays a critical role in OA progression, contributing to pain, cartilage degradation, and structural damage, emphasizing the inflammatory component alongside mechanical degeneration [1]. The knee is the most frequently affected joint symptomatically in OA [2]. Epidemiological studies conducted in various regions of the world have reported that 14–45% of individuals have symptomatic knee OA for their lifetime [3,4].

The incidence and progression of OA are influenced by a range of factors, including occupation, sports participation, musculoskeletal injuries, obesity, and gender, with significant societal and economic implications [5,6]. These factors highlight the widespread impact of knee OA and the importance of identifying effective treatment modalities.

The goals in the treatment of knee OA are to reduce pain and morning stiffness, maintain or restore joint range of motion and muscle strength, and decrease dependency in daily living activities. To achieve these goals, patient education, diet, physical therapy modalities, therapeutic exercises, medical and surgical treatment programs can be applied individually or in combination [7].

Physical therapy modalities are an important part of the treatment aimed at reducing pain, maintaining or improving joint range of motion, relieving spasms in affected muscles, or strengthening muscles [8]. Physical therapy modalities may also help patients better tolerate exercises. Although non-pharmacological strategies are crucial, fewer than 40% of individuals with knee OA participate in such treatments, highlighting that the adoption of evidence-based guidelines in clinical practice and rehabilitation remains inadequate [9].

Extracorporeal shock wave therapy (ESWT), pulsed electromagnetic field therapy (PEMF), and low-level laser therapy (LLLT) are distinct physical treatment modalities used for knee OA, each with different mechanisms of action. ESWT involves the application of high-energy sound waves to the tissue to reduce pain and inflammation. This method stimulates collagen production and increases fibroblast activity, promoting tissue repair [10]. It has been shown to not only enhance subchondral bone remodeling but also reduce the degradation of articular cartilage [10,11]. Several meta-analyses have demonstrated that ESWT can result in pain reduction and functional enhancement in knee OA [12,13,14]. PEMF utilizes magnetic fields to enhance healing. It aims to accelerate cellular metabolism and increase blood flow, thereby reducing pain and inflammation [15]. Research indicates that PEMF can alleviate pain and improve function, although some studies present mixed results [16,17,18]. LLLT employs low-level laser light to stimulate tissue healing. It enhances cellular energy production and exerts anti-inflammatory effects, which can help in pain reduction. Evidence supports that LLLT can reduce pain and improve function in knee OA, but the optimal application parameters are not yet fully clarified [19,20]. Overall, while all three methods show promise for treating knee OA, their effectiveness, treatment duration, and individual responses may vary.

A significant gap remains in the availability of high-quality randomized clinical trials for these physical therapy modalities. This study aims to evaluate and compare the short-term effects of ESWT, LLLT, and PEMF on pain, physical function, and quality of life in patients aged 40 to 70 years diagnosed with Kellgren–Lawrence grade 2–3 knee OA.

## 2. Materials and Methods

### 2.1. Study Design and Participants

The study included 120 patients who presented with knee pain to the PM&R outpatient clinic of Goztepe Prof Dr Suleyman Yalcin City Hospital and were diagnosed with Kellgren–Lawrence grade 2–3 knee OA based on the criteria of the ACR [21].

#### 2.1.1. Inclusion Criteria

Participants aged 40 to 70 years with knee pain persisting for more than six months and radiologically confirmed grade 2 or grade 3 knee osteoarthritis (OA) based on the Kellgren–Lawrence classification were included in the study. Eligibility required no history of physical therapy or regular non-steroidal anti-inflammatory drug (NSAID) use in the last six months to avoid residual effects from prior treatments. Participants were screened to ensure no other conditions, such as lumbar spine or hip pathologies, could refer pain to the knee, and only those with consistent pain levels for at least six months, indicating the absence of a remission phase, were enrolled. To ensure safe and regular participation, individuals had to be free from any conditions preventing physical activity and willing to adhere to the prescribed treatment programs.

#### 2.1.2. Exclusion Criteria

Participants were excluded if they had conditions impairing ambulation, such as severe mobility limitations, or a history of spinal stenosis or neurological disorders, which could interfere with study outcomes. Inflammatory or metabolic disorders leading to secondary OA were excluded to maintain a homogenous primary OA population. Patients who received intra-articular knee injections within the past year, or who used NSAIDs, paracetamol, or topical agents within the preceding week, were excluded to prevent any residual therapeutic effects from confounding the results. Additionally, individuals with a history of knee surgery were excluded, as such interventions could alter joint structure and biomechanics, potentially impacting treatment responses.

The randomized trial was conducted at Goztepe Prof Dr Suleyman Yalcin City Hospital, between January 2024 and June 2024. Physicians performing the pre- and post-evaluation and statistical analysis were blinded to the assignment of the groups. In the study, written informed consent was obtained from all participants prior to their inclusion. This process was conducted in accordance with the principles of the Declaration of Helsinki, ensuring that participants fully understood the nature, purpose, potential risks, and benefits of the study. Ethical approval was obtained from the Goztepe Prof Dr Suleyman Yalcin City Hospital Clinical Research Ethics Committee (approval no: 2020/0642). This study was registered in the ClinicalTrials.gov registry (registration no: NCT06717633) in accordance with international guidelines.

The sample size was computed by GPower 3.1. For the WOMAC total score, at least 16 individuals should be included in each group, when a mean difference of 9.1 between groups is considered significant with 80% power at the 95% confidence level (means 53.3 and 62.4; standard deviations 9.0 and 8.7) [22].

This study evaluates multiple outcomes without predefining a primary outcome, as pain, physical function, and quality of life are interrelated and equally prioritized in assessing the therapeutic impact on knee OA.

Adverse effects were defined as any worsening of knee OA symptoms, including increased pain, swelling, or stiffness, regardless of their relationship to the intervention. Participants were instructed to report any discomfort or unusual symptoms during or after treatment sessions, and the treating therapist actively inquired about potential side effects during each session.

Patients were assigned to groups utilizing a block randomization approach, as determined by the Randomizer Software (https://www.randomizer.org, accessed on 16 January 2024). Random allocation was carried out using sequentially numbered containers. Daily (3 × 10 repetitions each exercise) knee joint range of motion (ROM), stretching, isometric, and isotonic strengthening exercises were taught to all groups. Additionally, one group received ESWT, another received LLLT, and a third received PEMF. No analgesic or anti-inflammatory medications, including paracetamol or topical agents, were permitted during the study to avoid confounding effects. The evaluator, the person responsible for randomizing the patients, and the therapist administering the treatment were distinct individuals to minimize bias and ensure objectivity in the study.

**Figure 1:**
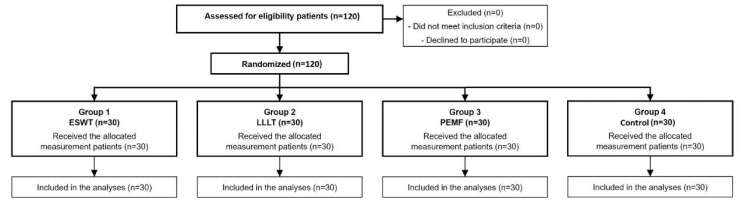
Flow chart of the study.

The flow chart of the study is shown in Figure 1.

### 2.2. Interventions

#### 2.2.1. Extracorporeal Shockwave Therapy

**Figure 2:**
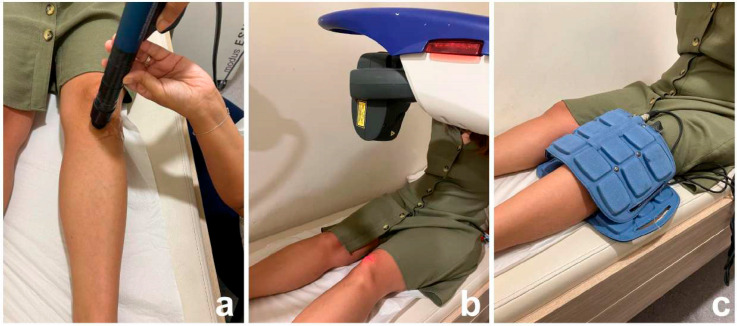
Application example of physical therapy modalities: (**a**) extracorporeal shock wave therapy (ESWT), (**b**) low-level laser therapy (LLLT), (**c**) pulsed electromagnetic field therapy (PEMF).

The ESWT was administered using an ESWT device (Modus ESWT Compact Model-Portable, Inceler Medical Ltd., Ankara, Turkey) once weekly over three consecutive weeks, resulting in a total of three sessions. A radial shockwave mode was chosen for the therapy. The treatment area was prepared by washing the skin. Participants were positioned lying on their back with the target knee bent at a 90° angle, while the physician stood on the same side as the treated limb. The physician identified tender points by palpating the patellofemoral and tibiofemoral borders. To reduce shock wave loss between the applicator and the skin, a gel was applied before ESWT delivery. During each session, participants received 3000 pulses at a 12 Hz frequency and pneumatic pressure of 2.5 bars [23] (Figure 2).

#### 2.2.2. Low-Level Laser Therapy

The MLS (Multiwave Locked System) laser is a specific type of low-level laser therapy (LLLT) that combines two different wavelengths of light—typically 808 nm (near-infrared) and 905 nm (mid-infrared)—to produce therapeutic effects. These wavelengths are delivered simultaneously in a synchronized manner, which is intended to enhance the overall effectiveness of the treatment. LLLT was conducted with the laser device (MLS Laser M6 Robotic Multiwave Lock System, Asalaser, Italy) with the use of protective goggles, and the treatment parameters were set as follows: 100% power, 3 Joules per treatment spot with 904 nm wavelength, and a treatment area of 20 cm^2^. The LLLT was applied to five points on the anterior part of the joint space, with each point treated for 3 min, totaling 15 min. A total of 8 sessions were applied for 4 weeks, 2 days a week [22]. Patients were informed that they might feel no sensation or a mild warmth in the treated area, which would dissipate immediately after the session (Figure 2).

#### 2.2.3. Pulsed Electromagnetic Field Therapy

For PEMF, the applicator of the device (Easy QS Magnetotherapy Portable Magnetotherapy Medical Device, Asalaser, Italy) was positioned to target the medial and lateral aspects of the knee. The treatment parameters were defined as follows: rectangular field shape, 30 Hz frequency, 10 mT intensity, and a duration of 20 min. A total of 8 sessions were applied for 4 weeks, 2 days a week [22]. Patients were informed that they would not feel any sensation during the treatment (Figure 2).

### 2.3. Outcome Measures

Measurements were performed baseline and immediately after 4 weeks of treatment.

#### 2.3.1. Visual Analog Scale (VAS)

Pain assessment in the study was conducted using the VAS, which ranges from 0 to 10. Patients were instructed to evaluate the pain they experienced. A score of “0” signified the absence of pain, whereas a score of “10” denoted the most intense pain they had ever encountered in their lifetime [24].

#### 2.3.2. Western Ontario and McMaster Universities Osteoarthritis Index (WOMAC)

The WOMAC OA Index is a questionnaire completed by the patient to assess pain, stiffness, and physical function related to knee and hip OA. It consists of 24 questions addressing pain, stiffness, and physical function. The index provides both a total score and separate subscores for each category. Higher scores indicate poorer outcomes in that category. The adaptation and validity study was conducted by Tüzün and colleagues [25].

#### 2.3.3. Short Form-36 (SF-36)

Among quality of life scales, the SF-36 is a generic scale that provides a broad assessment. It comprises 36 items that assess eight dimensions: physical functioning (PF), social functioning (SF), role limitations due to physical health (RLPH), role limitations due to emotional problems (RLEP), emotional well-being (EW), energy/fatigue (E), pain (P), general health (GH) and health change (HC). The adaptation and validity study was conducted by Koçyiğit and colleagues [26].

#### 2.3.4. Timed Up and Go Test (TUG)

This test is designed to assess dynamic balance and functional mobility. The test requires a chair, a stopwatch, and a 3 m walking space. It begins with the individual seated in a chair. Upon receiving the instruction, the individual stands up, walks 3 m at a regular speed, turns around, walks back to the starting point, and then sits down again. The time taken to complete the test is recorded in seconds for scoring purposes [27].

### 2.4. Statistical Analysis

Descriptive statistics for qualitative variables in the study were reported as frequency and %, while for quantitative variables were provided as mean, SD, minimum, and maximum values. A Shapiro–Wilk test was used to assess the alignment of quantitative variables with a normal distribution. Levene test was used for assess the homogeneity of variances. Relationships between qualitative variables were evaluated with Pearson chi-square or Fisher exact tests. A one-way ANOVA was employed comparing the means of three groups. A Kruskal–Wallis test was used to compare the medians of the three groups, and Dunn test was used in post hoc comparisons. A Friedman test was used to compare the means of more than two dependent groups. A post hoc analysis made with Bonferroni corrections. The statistical significance level was set as 0.05, and SPSS (Version 26.0. Armonk, NY, USA, IBM Corp.) was used in calculations.

## 3. Results

The characteristics of the participants are presented in Table 1. No significant difference was observed between the groups regarding age, gender, BMI, treatment side, and pain duration (*p* = 0.601; *p* = 0.361; *p* = 0.644; *p* = 0.191, *p* = 0.505, respectively).

**Figure 3:**
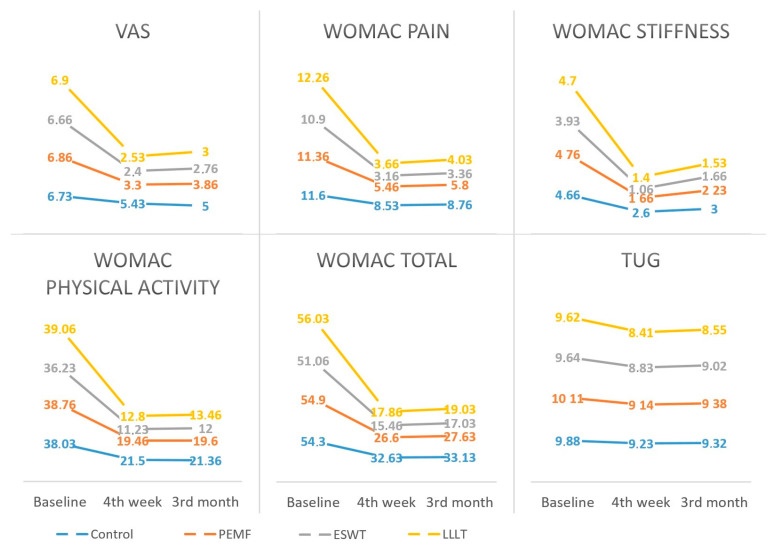
Comparison of scores between four groups (control, extracorporeal shock wave therapy (ESWT), low-level laser therapy (LLLT), pulsed electromagnetic field therapy (PEMF)).

No statistically significant difference was observed between the groups regarding pre-intervention VAS, WOMAC and subscores, SF-36 and subscores, and TUG means (*p* > 0.05 for each). It was determined that there was a statistically significant difference between the pre–post and 3rd month means for each group and score (*p* < 0.001 for each) (Figure 3) (Table 2).

A statistically significant difference was observed between groups pre–post and pre-3rd month mean changes in all parameters except TUG (for TUG: pre–post *p* = 0.275; pre-3rd month *p* = 0.246; post-3rd month *p* = 0.861; for all other parameters: pre–post and pre 3rd month *p* < 0.05; post-3rd month *p* > 0.05) (Table 3 and Table 4).

In post hoc analysis comparing the treatment modalities, the results indicated that the improvements in VAS, WOMAC pain, WOMAC physical function, WOMAC total, SF-36 PF, SF-36 RLEP, SF-36 E, SF-36 EW, SF-36 P scores were comparable between the ESWT and LLLT groups (*p* > 0.05). In contrast, the PEMF group exhibited significantly lower improvements in these parameters compared to both ESWT and LLLT (*p* < 0.001), and improvements in SF-36 RLPH and HC scores in the ESWT group were significantly higher than the LLLT group (*p* < 0.05); no statistically significant difference was observed between the PEMF and LLLT groups (*p* > 0.05), as summarized in Table 3 and Table 4.

No adverse effects were reported by participants during the study; however, this result may be influenced by underreporting or the short observation period.

## 4. Discussion

The purpose of this study was to compare the short-term effects of ESWT, LLLT, and PEMF on pain, physical function, and quality of life in patients with knee OA. It was observed that all three modalities led to significant improvements in pain, function and quality of life after 4 weeks, and these effects were more pronounced in the ESWT and LLLT groups.

Each therapy in this study—ESWT, LLLT, and PEMF—operates through distinct mechanisms. ESWT uses high-energy acoustic waves to enhance fibroblast activity, collagen production, angiogenesis, and subchondral bone remodeling, reducing inflammation and promoting tissue repair [10,11]. LLLT stimulates mitochondrial activity, increasing ATP synthesis, modulating inflammation, and enhancing blood flow and tissue healing [28]. PEMF induces microcurrents that stabilize membrane potential, boost cellular metabolism, increase nitric oxide production for vasodilation, and support cartilage repair by modulating chondrocyte activity [15,29].

While each modality shows promise, variations in therapeutic parameters (e.g., frequency, intensity, and duration) may significantly impact outcomes. The parameters used in this study align closely with those reported in the literature. For ESWT, the frequency (12 Hz), pressure (2.5 bars), and 3000 pulses per session are within the effective ranges of 10–15 Hz, 1.5–4 bars, and 1500–4000 pulses reported in prior studies [12]. Similarly, the LLLT parameters of 904 nm wavelength and 3 Joules per treatment point match the commonly cited effective ranges of 600–1000 nm and 3–5 J per point [30]. For PEMF, the frequency (30 Hz), intensity (10 mT), and session duration (20 min) are consistent with established protocols, which often use 10–50 Hz and 1–20 mT [29]. These alignments suggest that the study’s methodology is in line with evidence-based practices, but future research should explore further parameter optimization to maximize efficacy.

Pain reduction is a key concern for individuals seeking non-invasive medical treatment for symptom relief, as an immediate response to therapy can encourage continued treatment. In patients with knee OA, greater reductions in joint pain during movement are associated with improved physical function [31]. This study revealed that all three treatment modalities were effective in reducing pain levels, which corresponded with an increase in walking speed. In the literature, the effects of all three modalities on pain and functionality have been demonstrated by high-level evidence studies.

A recent systematic review and meta-analysis by Rayegani et al. [32] reported significant pain reduction in knee OA patients treated with LLLT. Montes et al. [33] demonstrated that LLLT, when combined with exercise for quadriceps strengthening, is a reliable and efficient method for alleviating knee pain. A recent experimental study suggests that both the exercise program and LLLT are effective in avoiding cartilage breakdown and modulating inflammatory responses related to knee OA [34]. A systematic review indicates that LLLT application prior to exercise results in performance-enhancing and shielding benefits for skeletal muscles [35], while administering it after injury helps safeguard cells from secondary damage due to its anti-inflammatory and oxidative stress-reducing properties [36]. Martin Bjørn Stausholm et al.’s [28] meta-analysis revealed that recommended doses of LLLT significantly reduced pain and disability in knee OA compared to the placebo, with sustained effects observed up to 12 weeks post-treatment; however, the heterogeneity of the studies, due to the use of non-recommended doses and varying parameters, limits the overall consistency of the findings.

Li et al.’s [12] meta-analysis highlights the superiority of ESWT over placebo and physical therapy in treating knee OA. The study found that ESWT not only provides greater pain relief, but also enhances knee joint mobility and may reduce the Lequesne index (LI) and WOMAC scores. Peiyuan Tang et al.’s [23] recent umbrella review including eight meta-analyses with high-quality ratings assessed the efficacy of ESWT compared to non-ESWT treatments for knee OA. The review concludes that ESWT is an effective treatment for alleviating pain and improving function in knee OA.

Tong et al. [37] conducted a meta-analysis demonstrating that PEMF significantly relieves pain, reduces stiffness, and enhances physical function in OA patients compared to other conservative treatments. Iannitti et al. [29] found that PEMF significantly improves pain, stiffness, and physical function in elderly patients with knee OA. Chen et al.’s [38] review and meta-analysis found that PEMF enhances physical function in knee OA patients, although it does not significantly alleviate pain or stiffness. The study supports PEMF as an adjunctive treatment but calls for further high-quality trials to optimize its application and parameters.

Despite the numerous studies in the literature in recent years and the increasing evidence, studies directly comparing these three treatment modalities with one another are relatively scarce. Mona El Naggar et al. [39] compared ESWT and LLLT for knee OA, finding that both therapies significantly improved pain and functional outcomes. However, neither ESWT nor LLLT was found to be superior to the other, as both showed comparable effects in relieving symptoms and enhancing function. A retrospective study by Wei Li et al. [40] reported that ESWT may relieve symptoms of knee OA more effectively and be safe compared with laser therapy. In this study, consistent with the findings of the two referenced studies, both ESWT and LLLT demonstrated comparable effectiveness across most parameters; however, ESWT exhibited superior outcomes, specifically in the SF-36 RLPH and HC subscores. Similarly, the literature indicates that evidence supporting ESWT and LLLT is more robust compared to PEMF. It is possible that the regenerative and anti-inflammatory effects of ESWT and LLLT at the cellular level may be more pronounced. In a study by Tomazoni et al., it was found that LLLT reduced inflammatory markers and cytokines in rats with knee OA more effectively than NSAIDs, despite both treatments similarly lowering levels of inflammatory cells and metalloproteinases [41]. Zhao et al. [42] explored the mechanism of ESWT for knee OA in rabbits, finding that ESWT significantly reduced nitric oxide (NO) levels and chondrocyte apoptosis in the knee joint’s synovial cavity. In contrast to this study, Elboim-Gabyzon et al.’s [22] study demonstrated that PEMF was more effective than LLLT in reducing pain and enhancing function in people with knee OA. This may be due to the lower laser therapy wavelength applied or that the laser therapy device used in this study was a special type of LLLT, the multiwave locked system.

These therapies’ clinical applicability depends not only on efficacy but also on cost, availability, and patient preferences. ESWT is typically more expensive due to costly equipment and required specialized training, while LLLT is more cost-effective and accessible, making it suitable for resource-limited settings. PEMF devices vary in cost but are often less available. Patient preferences also influence choice; ESWT may cause mild discomfort, whereas LLLT and PEMF are non-invasive, painless, and generally well-tolerated, appealing to those seeking gentle treatment options.

### Strengths and Limitations

This study evaluates only the short-term effects (4 weeks) of ESWT, LLLT, and PEMF on knee OA. Long-term follow-up studies assessing outcomes at 6 and 12 months are necessary to evaluate the durability of therapeutic effects and guide clinical decisions. Although the sample size of 120 patients provides sufficient power for our analysis, the relatively small size of each treatment group (*n* = 30) may limit the generalizability of the results. Future studies should consider larger participant groups to enhance the reliability and applicability of findings.

The outcomes of LLLT and PEMF are influenced by therapeutic parameters such as dose, frequency, and intensity. Future studies should explore how varying these parameters impacts treatment efficacy to optimize protocols for individual patient needs. While ESWT and LLLT demonstrated comparable efficacy, considerations such as cost, device availability, and patient preference play crucial roles in clinical decision-making. For instance, ESWT may be less accessible due to higher costs and limited device availability, whereas LLLT may offer a more cost-effective option. Future studies should evaluate patient-reported preferences and cost-effectiveness for each therapy. Also, more pronounced physical sensations associated with ESWT, such as the potential placebo effect due to operator influence, the visible laser light cursor, and the slight warmth experienced during LLLT may contribute to these results, given that PEMF does not evoke any distinct sensory differences compared to ESWT and LLLT.

Furthermore, the study has a number of strengths. Although many studies have been conducted in the literature on ESWT, LLLT and PEMF, a head-to-head comparison of these three non-invasive and popular modalities has been made for the first time, to our knowledge. Also, while existing literature predominantly examines pain and function, often neglecting their impact on quality of life, this study encompasses the effects of all three modalities on the quality of life in individuals with knee OA. Further research is needed to investigate their long-term efficacy, mechanisms of action, and cost-effectiveness. Additionally, future studies should incorporate larger sample sizes, long-term follow-ups, and strategies to monitor and ensure adherence to interventions.

## 5. Conclusions

This study shows that in the short term, ESWT and LLLT are more effective than PEMF in reducing pain, augmenting physical function, and advancing quality of life in patients with knee OA. However, PEMF also shows significant efficacy and may be a valuable adjunctive treatment option. Long-term studies are essential to better evaluate the durability of these effects and refine treatment strategies.

## Figures and Tables

**Table 1:** Demographic data for groups.

	Control (*n* = 30)	ESWT (*n* = 30)	LLLT (*n* = 30)	PEMF (*n* = 30)	*p*
Age (years)	60.07 ± 8.05 60(46–74)	60.43 ± 7.49 61 (45–69)	62.33 ± 9.53 64 (44–69)	60.66 ± 9.20 65.5 (42–69)	0.601 ^1^
Gender (F/M)	26 (86.7) 4 (13.3%)	29 (96.7%)/ 1 (3.3%)	25 (83.3%)/ 5 (16.7%)	28 (93.3%)/ 2 (6.7%)	0.361 ^2^
BMI (kg/m^2^)	28.77 ± 3.82 29.13 (20.42–37.11)	29.50 ± 2.74 29.37 (24.09–33.20)	28.84 ± 4.68 29.34 (20.42–37.11)	29.84 ± 3.78 29.83 (22.27–37.11)	0.644 ^3^
Treatment side (right/left)	17 (56.7%) 13 (43.3%)	15 (50%)/ 15 (50%)	11 (36.7%)/ 19 (63.3%)	19 (63.3%)/ 11 (36.7%)	0.197 ^4^
Pain duration (years)	4.36 ± 5.16 2 (1–20)	3.90 ± 2.35 3 (1–8)	3.48 ± 3.05 3 (1–15)	4.63 ± 5.74 2 (1–20)	0.505 ^1^

ESWT: extracorporeal shockwave therapy, LLLT: low-level laser therapy, PEMF: pulsed electromagnetic field therapy. Descriptive statistics are presented as mean ± standard deviation and median [min–max] for continuous variables, and *n* (%) for categorical variables. ^1^: Kruskal–Wallis, ^2^: Fisher exact test, ^3^: one-way ANOVA, ^4^: Pearson chi-square.

**Table 2:** Comparison of pre- and 3rd month change means of variables.

Group		VAS Change	WOMAC Pain Change	WOMAC Stiffness Change	WOMAC Physical Activity Change	WOMAC Total Change	SF-36 PF Change	SF-36 RLPH Change	SF-36 RLEP Change	SF-36 E Change	SF-36 EW Change	SF-36 SF Change	SF-36 P Change	SF-36 GH Change	SF-36 HC	TUG Change
Control	Z	−4.01 ^b^	−4.655 ^b^	−4.363 ^b^	−4.591 ^b^	−4.683 ^b^	−4.566 ^c^	−4.190 ^c^	−3.862 ^c^	−4.392 ^c^	−4.429 ^c^	−4.572 ^c^	−4.445 ^c^	−4.449 ^c^	−4.562 ^c^	−3.932 ^b^
	*p*	<0.001	<0.001	<0.001	<0.001	<0.001	<0.001	<0.001	<0.001	<0.001	<0.001	<0.001	<0.001	<0.001	<0.001	<0.001
LLLT	Z	−4.671 ^b^	−4.635 ^b^	−4.581 ^b^	−4.629 ^b^	−4.625 ^b^	−4.558 ^c^	−4.479 ^c^	−4.514 ^c^	−4.539 ^c^	−4.465 ^c^	−4.503 ^c^	−4.554 ^c^	−4.559 ^c^	−3.897 ^c^	−3.975 ^b^
	*p*	<0.001	<0.001	<0.001	<0.001	<0.001	<0.001	<0.001	<0.001	<0.001	<0.001	<0.001	<0.001	<0.001	<0.001	<0.001
PEMF	Z	−4.658 ^b^	−4.467 ^b^	−4.383 ^b^	−4.626 ^b^	−4.624 ^b^	−4.183 ^c^	−4.383 ^c^	−4.339 ^c^	−4.095 ^c^	−4.462 ^c^	−4.396 ^c^	−4.271 ^c^	−4.130 ^c^	−4.217 ^c^	−3.887 ^b^
	*p*	<0.001	<0.001	<0.001	<0.001	<0.001	<0.001	<0.001	<0.001	<0.001	<0.001	<0.001	<0.001	<0.001	<0.001	<0.001
ESWT	Z	−4.771 ^b^	−4.715 ^b^	−4.406 ^b^	−4.784 ^b^	−4.784 ^b^	−4.795 ^c^	−4.789 ^c^	−4.805 ^c^	−4.802 ^c^	−4.727 ^c^	−4.708 ^c^	−4.816 ^c^	−4.807 ^c^	−4.832 ^c^	−4.786 ^b^
	*p*	<0.001	<0.001	<0.001	<0.001	<0.001	<0.001	<0.001	<0.001	<0.001	<0.001	<0.001	<0.001	<0.001	<0.001	<0.001

ESWT: extracorporeal shockwave therapy, LLLT: low-level laser therapy, PEMF: pulsed electromagnetic field therapy, VAS: visual analog scale, WOMAC: Western Ontario and McMaster Universities Osteoarthritis Index, TUG: Timed Up and Go, SF-36: short form-36, PF: physical functioning, RLPH: role limitations due to physical health, RLEP: role limitations due to emotional problems, E: energy/fatigue, EW: emotional well-being, SF: social functioning, P: pain, GH: general health, HC: health change. Wilcoxon signed rank test was used. ^b^: based on positive ranks, ^c^: based on negative ranks. All *p* values were *p* < 0.001 (statistically significant).

**Table 3:** Comparison of pre–post and 3rd month change means of VAS, WOMAC, and TUG between groups.

	Group	Mean	Median	Std. Deviation	Minimum	Maximum	*p*
VAS Pre–post	Control	1.30	1.00	0.87	0.00	3.00	<0.001 Control vs. PEMF ≤ 0.001 Control vs. ESWT ≤ 0.001 Control vs. LLLT ≤ 0.001 PEMF vs. ESWT = 0.048 PEMF vs. LLLT = 0.030 ESWT vs. LLLT = 0.843
	LLLT	4.36	4.00	0.96	2.00	6.00	
	PEMF	3.56	4.00	1.19	1.00	5.00	
	ESWT	4.26	4.00	0.86	1.00	5.00	
VAS Pre-3rd month	Control	1.73	2.00	1.04	0.00	4.00	<0.001 Control vs. PEMF = 0.001 Control vs. ESWT ≤ 0.001 Control vs. LLLT ≤ 0.001 PEMF vs. ESWT = 0.007 PEMF vs. LLLT = 0.011 ESWT vs. LLLT = 0.887
	LLLT	3.90	4.00	1.06	1.00	6.00	
	PEMF	3.00	3.00	1.43	1.00	6.00	
	ESWT	3.90	4.00	0.88	2.00	5.00	
VAS Post-3rd month	Control	0.43	0.00	0.56	0.00	2.00	<0.001 Control vs. PEMF ≤ 0.001 Control vs. ESWT ≤ 0.001 Control vs. LLLT ≤ 0.001 PEMF vs. ESWT = 0.307 PEMF vs. LLLT = 0.524 ESWT vs. LLLT = 0.702
	LLLT	−0.46	0.00	0.68	−2.00	1.00	
	PEMF	−0.56	−1.00	0.93	−3.00	2.00	
	ESWT	−0.36	0.00	0.71	−2.00	1.00	
WOMAC pain Pre–post	Control	3.06	2.00	1.76	0.00	8.00	<0.001 Control vs. PEMF ≤ 0.001 Control vs. ESWT ≤ 0.001 Control vs. LLLT ≤ 0.001 PEMF vs. ESWT = 0.043 PEMF vs. LLLT = 0.002 ESWT vs. LLLT = 0.313
	LLLT	8.60	9.00	2.72	2.00	13.00	
	PEMF	5.90	6.00	3.15	−3.00	12.00	
	ESWT	7.73	8.00	2.69	4.00	14.00	
WOMAC pain Pre-3rd month	Control	2.83	2.50	1.91	0.00	8.00	<0.001 Control vs. PEMF = 0.001 Control vs. ESWT ≤ 0.001 Control vs. LLLT ≤ 0.001 PEMF vs. ESWT = 0.026 PEMF vs. LLLT = 0.003 ESWT vs. LLLT = 0.434
	LLLT	8.23	8.00	2.80	2.00	14.00	
	PEMF	5.56	5.50	3.21	−4.00	13.00	
	ESWT	7.53	7.50	2.66	3.00	14.00	
WOMAC Post-3rd month	Control	−0.23	0.00	0.62	−1.00	1.00	0.654
	LLLT	−0.36	0.00	0.80	−2.00	2.00	
	PEMF	−0.33	−0.50	0.88	−2.00	2.00	
	ESWT	−0.20	0.00	0.71	−1.00	2.00	
WOMAC stiffness Pre–post	Control	2.06	2.00	1.22	0.00	4.00	0.005 Control vs. PEMF = 0.031 Control vs. ESWT = 0.022 Control vs. LLLT ≤ 0.001 PEMF vs. ESWT = 0.896 PEMF vs. LLLT = 0.176 ESWT vs. LLLT = 0.221
	LLLT	3.30	3.00	1.34	0.00	5.00	
	PEMF	3.10	3.00	2.32	0.00	12.00	
	ESWT	2.86	3.00	1.27	0.00	5.00	
WOMAC stiffness Pre-3rd month	Control	1.66	1.00	1.29	0.00	4.00	0.001 Control vs. PEMF = 0.113 Control vs. ESWT = 0.050 Control vs. LLLT ≤ 0.001 PEMF vs. ESWT = 0.819 PEMF vs. LLLT = 0.014 ESWT vs. LLLT = 0.026
	LLLT	3.16	3.00	1.39	0.00	6.00	
	PEMF	2.53	2.00	2.45	−1.00	13.00	
	ESWT	2.26	3.00	1.38	−1.00	5.00	
WOMAC stiffness Post−3rd month	Control	−0.40	0.00	0.72	−2.00	1.00	0.140
	LLLT	−0.13	0.00	0.57	−2.00	1.00	
	PEMF	−0.56	−1.00	0.89	−3.00	1.00	
	ESWT	−0.60	0.00	1.32	−5.00	1.00	
WOMAC physical activity Pre–post	Control	16.53	15.00	8.15	1.00	30.00	<0.001 Control vs. PEMF = 0.309 Control vs. ESWT ≤ 0.001 Control vs. LLLT ≤ 0.001 PEMF vs. ESWT = 0.013 PEMF vs. LLLT = 0.002 ESWT vs. LLLT = 0.580
	LLLT	26.26	28.50	8.23	9.00	41.00	
	PEMF	19.30	18.50	8.27	6.00	38.00	
	ESWT	25.00	24.00	7.66	6.00	38.00	
WOMAC physical activity Pre-3rd month	Control	16.66	15.50	7.92	1.00	30.00	<0.001 Control vs. PEMF = 0.355 Control vs. ESWT = 0.001 Control vs. LLLT ≤ 0.001 PEMF vs. ESWT = 0.021 PEMF vs. LLLT = 0.004 ESWT vs. LLLT = 0.526
	LLLT	25.60	28.50	8.82	7.00	41.00	
	PEMF	19.16	18.50	8.63	3.00	38.00	
	ESWT	24.23	23.00	7.48	6.00	38.00	
WOMAC physical activity Post-3rd month	Control	0.13	0.00	2.89	−8.00	6.00	0.793
	LLLT	−0.66	0.00	2.17	−5.00	5.00	
	PEMF	−0.13	0.00	3.22	−12.00	9.00	
	ESWT	−0.7667	0.00	2.52	−11.00	4.00	
WOMAC total Pre–post	Control	21.66	19.00	9.48	5.00	39.00	<0.001 Control vs. PEMF = 0.050 Control vs. ESWT ≤ 0.001 Control vs. LLLT ≤ 0.001 PEMF vs. ESWT = 0.024 PEMF vs. LLLT = 0.002 ESWT vs. LLLT = 0.382
	LLLT	38.16	40.00	10.81	12.00	55.00	
	PEMF	28.30	28.50	11.54	5.00	54.00	
	ESWT	35.60	33.50	10.88	11.00	57.00	
WOMAC total Pre-3rd month	Control	21.16	21.00	9.49	4.00	38.00	<0.001 Control vs. PEMF = 0.050 Control vs. ESWT ≤ 0.001 Control vs. LLLT ≤ 0.001 PEMF vs. ESWT = 0.034 PEMF vs. LLLT = 0.002 ESWT vs. LLLT = 0.333
	LLLT	37.00	39.50	11.30	11.00	53.00	
	PEMF	27.26	28.00	11.92	4.00	52.00	
	ESWT	34.03	32.50	10.16	11.00	56.00	
WOMAC total Post-3rd month	Control	−0.50	−1.00	3.07	−8.00	7.00	0.829
	LLLT	−1.16	−1.00	2.53	−6.00	6.00	
	PEMF	−1.03	−1.00	3.81	−13.00	12.00	
	ESWT	−1.56	−1.00	3.23	−12.00	4.00	
TUG Pre–post	Control	0.65	0.60	0.59	0.00	2.63	0.275
	LLLT	1.21	0.92	1.13	0.00	4.45	
	PEMF	0.96	0.90	0.72	−0.18	3.13	
	ESWT	0.80	0.66	0.64	0.08	2.30	
TUG Pre-3rd month	Control	0.56	0.55	0.60	−0.10	2.63	0.246
	LLLT	1.06	0.90	1.11	−1.30	4.00	
	PEMF	0.72	0.70	0.70	−0.85	2.00	
	ESWT	0.62	0.39	0.58	0.01	2.30	
TUG Post-3rd month	Control	−0.09	0.00	0.20	−0.80	0.40	0.861
	LLLT	−0.14	−0.10	0.58	−1.70	1.50	
	PEMF	−0.24	0.00	0.47	−1.70	0.16	
	ESWT	−0.18	0.00	0.28	−0.90	0.02	

ESWT: extracorporeal shockwave therapy, LLLT: low-level laser therapy, PEMF: pulsed electromagnetic field therapy, VAS: visual analog scale, WOMAC: Western Ontario and McMaster Universities Osteoarthritis Index, TUG: timed up and go. Descriptive statistics are presented as mean ± standard deviation and median [min–max] for continuous variables.

**Table 4:** Comparison of pre–post and 3rd month change means of SF-36 between groups.

	Group	Mean	Median	Std. Deviation	Minimum	Maximum	*p*
SF-36 PF Pre–post	Control	13.16	12.50	8.25	0.00	30.00	<0.001 Control vs. PEMF = 0.039 Control vs. ESWT ≤ 0.001 Control vs. LLLT ≤ 0.001 PEMF vs. ESWT ≤ 0.001 PEMF vs. LLLT ≤ 0.001 ESWT vs. LLLT = 0.632
	LLLT	39.33	40.00	13.11	0.00	60.00	
	PEMF	21.50	20.00	17.47	−15.00	60.00	
	ESWT	37.33	35.00	11.65	5.00	60.00	
SF-36 PF Pre-3rd month	Control	10.33	10.00	8.60	−5.00	30.00	<0.001 Control vs. PEMF = 0.050 Control vs. ESWT ≤ 0.001 Control vs. LLLT ≤ 0.001 PEMF vs. ESWT ≤ 0.001 PEMF vs. LLLT ≤ 0.001 ESWT vs. LLLT = 0.819
	LLLT	35.50	35.00	13.15	0.00	60.00	
	PEMF	18.16	15.00	15.94	−10.00	55.00	
	ESWT	34.66	35.00	12.17	5.00	60.00	
SF-36 PF Post-3rd month	Control	−2.83	0.00	4.08	−15.00	0.00	0.829
	LLLT	−3.83	0.00	5.03	−20.00	0.00	
	PEMF	−3.33	0.00	6.06	−20.00	5.00	
	ESWT	−2.66	0.00	3.88	−15.00	0.00	
SF-36 RLPH Pre–post	Control	24.89	25.00	20.20	−45.00	50.00	<0.001 Control vs. PEMF ≤ 0.001 Control vs. ESWT ≤ 0.001 Control vs. LLLT = 0.001 PEMF vs. ESWT = 0.090 PEMF vs. LLLT = 0.349 ESWT vs. LLLT = 0.008
	LLLT	48.33	50.00	22.67	0.00	100.00	
	PEMF	55.00	50.00	31.07	0.00	100.00	
	ESWT	68.61	75.00	23.02	25.00	100.00	
SF-36 RLPH Pre-3rd month	Control	21.89	25.00	17.42	−25.00	50.00	<0.001 Control vs. PEMF ≤ 0.001 Control vs. ESWT ≤ 0.001 Control vs. LLLT ≤ 0.001 PEMF vs. ESWT = 0.089 PEMF vs. LLLT = 0.652 ESWT vs. LLLT = 0.028
	LLLT	45.83	50.00	22.01	0.00	100.00	
	PEMF	50.00	47.50	30.51	0.00	100.00	
	ESWT	62.11	55.00	23.23	25.00	100.00	
SF-36 RLPH Post-3rd month	Control	−3.00	0.00	6.64	−15.00	20.00	0.283
	LLLT	−2.50	0.00	3.65	−10.00	0.00	
	PEMF	−5.00	0.00	6.56	−20.00	0.00	
	ESWT	−6.50	−2.50	8.00	−25.00	0.00	
SF-36 RLEP Pre–post	Control	33.11	33.30	19.62	0.00	66.70	<0.001 Control vs. PEMF = 0.004 Control vs. ESWT ≤ 0.001 Control vs. LLLT ≤ 0.001 PEMF vs. ESWT = 0.082 PEMF vs. LLLT = 0.060 ESWT vs. LLLT = 0.885
	LLLT	60.58	66.70	27.75	0.00	100.00	
	PEMF	47.80	33.40	27.23	0.00	100.00	
	ESWT	60.25	66.70	17.54	33.30	100.00	
SF-36 RLEP Pre-3rd month	Control	29.78	33.30	26.77	−33.40	66.70	<0.001 Control vs. PEMF = 0.016 Control vs. ESWT ≤ 0.001 Control vs. LLLT ≤ 0.001 PEMF vs. ESWT = 211 PEMF vs. LLLT = 0.074 ESWT vs. LLLT = 0.594
	LLLT	57.68	66.70	28.57	0.00	100.00	
	PEMF	43.80	33.40	29.12	−33.30	100.00	
	ESWT	53.60	66.70	22.21	0.00	100.00	
SF-36 RLEP Post-3rd month	Control	−3.33	0.00	16.03	−33.40	33.40	0.832
	LLLT	−2.89	0.00	12.65	−33.40	33.30	
	PEMF	−4.00	0.00	14.18	−33.40	33.30	
	ESWT	−6.65	0.00	13.52	−33.30	0.00	
SF-36 EPre–post	Control	19.03	20.00	12.76	−4.00	45.00	<0.001 Control vs. PEMF = 0.050 Control vs. ESWT ≤ 0.001 Control vs. LLLT ≤ 0.001 PEMF vs. ESWT = 0.010 PEMF vs. LLLT = 0.009 ESWT vs. LLLT = 0.981
	LLLT	36.16	35.00	14.24	0.00	65.00	
	PEMF	25.36	25.00	17.81	−19.00	55.00	
	ESWT	35.73	35.00	9.14	20.00	50.00	
SF-36 E Pre-3rd month	Control	16.86	17.50	13.02	−9.00	40.00	<0.001 Control vs. PEMF = 0.146 Control vs. ESWT ≤ 0.001 Control vs. LLLT ≤ 0.001 PEMF vs. ESWT = 0.005 PEMF vs. LLLT = 0.001 ESWT vs. LLLT = 0.602
	LLLT	34.16	35.00	13.90	−10.00	65.00	
	PEMF	22.03	20.00	17.01	−19.00	55.00	
	ESWT	32.83	35.00	9.06	10.00	50.00	
SF-36 E Post-3rd month	Control	−2.16	0.00	2.84	−10.00	0.00	0.565
	LLLT	−2.00	0.00	5.34	−20.00	10.00	
	PEMF	−3.33	0.00	5.30	−15.00	5.00	
	ESWT	−2.90	0.00	3.92	−15.00	0.00	
SF-36 EW Pre–post	Control	17.85	16.00	12.73	0.00	48.00	<0.001 Control vs. PEMF = 0.001 Control vs. ESWT ≤ 0.001 Control vs. LLLT ≤ 0.001 PEMF vs. ESWT = 0.122 PEMF vs. LLLT = 1.000 ESWT vs. LLLT = 0.861
	LLLT	35.93	36.00	13.28	0.00	56.00	
	PEMF	31.13	34.00	14.14	0.00	60.00	
	ESWT	35.80	40.00	12.62	5.50	52.00	
SF-36 EW Pre-3rd month	Control	14.25	15.00	10.99	−6.50	36.00	<0.001 Control vs. PEMF = 0.003 Control vs. ESWT ≤ 0.001 Control vs. LLLT ≤ 0.001 PEMF vs. ESWT = 0.076 PEMF vs. LLLT = 0.036 ESWT vs. LLLT = 0.727
	LLLT	34.10	36.00	13.61	0.00	52.00	
	PEMF	26.33	26.00	14.81	0.00	60.00	
	ESWT	32.33	36.00	14.27	−6.50	52.00	
SF-36 EW Post-3rd month	Control	−3.60	0.00	6.06	−24.00	0.00	0.901
	LLLT	−2.10	0.00	3.29	−12.00	0.00	
	PEMF	−4.80	0.00	9.06	−32.00	0.00	
	ESWT	−3.46	0.00	4.54	−12.00	0.00	
SF-36 SF Pre–post	Control	21.08	25.00	11.32	0.00	50.00	0.001 Control vs. PEMF = 0.012 Control vs. ESWT = 0.001 Control vs. LLLT ≤ 0.001 PEMF vs. ESWT = 0.421 PEMF vs. LLLT = 0.211 ESWT vs. LLLT = 0.655
	LLLT	34.53	37.50	14.52	0.00	50.00	
	PEMF	29.70	25.00	14.62	0.00	50.00	
	ESWT	32.97	37.50	17.09	−12.50	62.50	
SF-36 SF Pre-3rd month	Control	18.83	22.50	12.92	−20.00	45.00	0.025 Control vs. PEMF = 0.174 Control vs. ESWT = 0.004 Control vs. LLLT = 0.023 PEMF vs. ESWT = 0.130 PEMF vs. LLLT = 0.361 ESWT vs. LLLT = 0.549
	LLLT	27.41	23.75	13.46	0.00	57.50	
	PEMF	23.41	22.50	16.22	−20.00	45.00	
	ESWT	30.08	25.00	12.39	12.50	55.00	
SF-36 SF Post-3rd month	Control	−9.96	−11.00	13.02	−30.00	15.00	0.056
	LLLT	−9.61	−7.50	9.06	−30.00	5.00	
	PEMF	−15.00	−17.50	8.01	−30.00	2.50	
	ESWT	−14.05	−17.50	10.60	−30.00	2.50	
SF-36 P Pre–post	Control	21.33	22.50	12.74	−20.00	45.00	0.003 Control vs. PEMF = 0.122 Control vs. ESWT ≤ 0.001 Control vs. LLLT = 0.021 PEMF vs. ESWT = 0.033 PEMF vs. LLLT = 0.450 ESWT vs. LLLT = 0.170
	LLLT	30.00	27.50	13.47	0.00	57.50	
	PEMF	26.58	22.50	17.16	−20.00	55.00	
	ESWT	34.66	32.50	11.07	12.50	55.00	
SF-36 P Pre-3rd month	Control	18.83	22.50	12.92	−20.00	45.00	0.025 Control vs. PEMF = 0.174 Control vs. ESWT = 0.004 Control vs. LLLT = 0.023 PEMF vs. ESWT = 0.130 PEMF vs. LLLT = 0.361 ESWT vs. LLLT = 0.549
	LLLT	27.41	23.75	13.46	0.00	57.50	
	PEMF	23.41	22.50	16.22	−20.00	45.00	
	ESWT	30.08	25.00	12.39	12.50	55.00	
SF-36 P Post-3rd month	Control	−2.50	0.00	4.64	−12.50	0.00	0.566
	LLLT	−2.58	0.00	4.22	−10.00	0.00	
	PEMF	−3.16	0.00	6.85	−22.50	0.00	
	ESWT	−4.58	0.00	6.79	−22.50	0.00	
SF-36 GH Pre–post	Control	14.25	15.00	9.44	0.00	35.00	0.026 Control vs. PEMF = 0.876 Control vs. ESWT = 0.240 Control vs. LLLT = 0.007 PEMF vs. ESWT = 0.308 PEMF vs. LLLT = 0.011 ESWT vs. LLLT = 0.127
	LLLT	21.50	20.00	10.09	0.00	40.00	
	PEMF	14.75	15.00	11.64	0.00	40.00	
	ESWT	17.16	15.00	8.37	5.00	35.00	
SF-36 GH Pre-3rd month	Control	13.08	12.50	9.36	−2.50	30.00	0.042 Control vs. PEMF = 0.878 Control vs. ESWT = 0.251 Control vs. LLLT = 0.011 PEMF vs. ESWT = 0.321 PEMF vs. LLLT = 0.017 ESWT vs. LLLT = 0.165
	LLLT	19.66	20.00	9.46	0.00	40.00	
	PEMF	13.58	12.50	10.90	0.00	35.00	
	ESWT	16.00	15.00	8.74	5.00	35.00	
SF-36 GH Post-3rd month	Control	−1.16	0.00	2.84	−10.00	5.00	0.811
	LLLT	−1.83	0.00	3.07	−10.00	0.00	
	PEMF	−1.16	0.00	2.84	−10.00	5.00	
	ESWT	−1.16	0.00	2.15	−5.00	0.00	
SF-36 HC Pre–post	Control	25.00	25.00	12.79	0.00	50.00	0.002 Control vs. PEMF = 0.864 Control vs. ESWT = 0.001 Control vs. LLLT = 0.362 PEMF vs. ESWT = 0.001 PEMF vs. LLLT = 0.459 ESWT vs. LLLT = 0.014
	LLLT	30.83	25.00	25.15	−25.00	75.00	
	PEMF	27.00	25.00	17.30	0.00	50.00	
	ESWT	41.66	50.00	11.98	25.00	50.00	
SF-36 HC Pre-3rd month	Control	22.66	25.00	12.50	0.00	50.00	0.001 Control vs. PEMF = 0.891 Control vs. ESWT ≤ 0.001 Control vs. LLLT = 0.197 PEMF vs. ESWT ≤ 0.001 PEMF vs. LLLT = 0.249 ESWT vs. LLLT = 0.017
	LLLT	28.83	25.00	24.83	−25.00	75.00	
	PEMF	23.66	25.00	14.67	0.00	50.00	
	ESWT	39.33	45.00	13.56	15.00	55.00	
SF-36 HC Post-3rd month	Control	−2.33	0.00	4.68	−15.00	5.00	0.794
	LLLT	−2.00	0.00	3.37	−10.00	5.00	
	PEMF	−3.33	0.00	6.47	−15.00	15.00	
	ESWT	−2.33	0.00	4.49	−10.00	5.00	

ESWT: extracorporeal shockwave therapy, LLLT: low-level laser therapy, PEMF: pulsed electromagnetic field therapy, SF-36: short form-36, PF: physical functioning, RLPH: role limitations due to physical health, RLEP: role limitations due to emotional problems, E: energy/fatigue, EW: emotional well-being, SF: social functioning, P: pain, GH: general health, HC: health change.

## Data Availability

The data are available to consult.
